# Opioid Knowledge and Prescribing Habits at a Large Tertiary Care Academic Center

**DOI:** 10.7759/cureus.27843

**Published:** 2022-08-10

**Authors:** Bajaj Prempreet, Megan Brennan, Gregory Grigoropoulos, Adam Hintz, Satyum Parikh, Neha Shah, Amy Wozniak

**Affiliations:** 1 Orthopaedic Surgery and Rehabilitation, Loyola University Medical Center, Maywood, USA; 2 Physical Medicine & Rehabilitation, Loyola University Chicago Stritch School of Medicine, Maywood, USA; 3 Physical Medicine and Rehabilitation, Marianjoy Rehabilitation Hospital, Wheaton, USA; 4 Statistics, Clinical Research Office Biostatistics Collaborative Core, Loyola University Chicago Health Sciences Division Center for Translational Research and Education, Maywood, USA

**Keywords:** monitoring program, prescription drug, urine drug screen, medical education, pain management, opioids

## Abstract

Background: Opioids are commonly prescribed medications for pain management with high risks associated with chronic use. The inherent risk associated with opioids is worsened by variable prescribing practices used by prescribers. In the midst of the “opioid epidemic,” perceptions of opioid prescription among healthcare practitioners have not been widely investigated.

Objective: This study aimed to explore the opinions, experiences, and habits of prescribers as well as other healthcare personnel involved in the administration of opioids at an academic medical center.

Methods: Questions were shared through an online survey format, answerable in Likert scale scores from 1 to 5*, *and categorized into three domains; prescribing habits/management, education, and risk stratification.

Results: A total of 638 survey responses were collected comprising 130 physicians (21%), 44 residents and fellows (6.9%), 53 physician assistants and nurse practitioners (8.31%), 18 pharmacists (2.82%), 85 medical students (13.32%), and 308 nurses (48.28%). Collected responses revealed a weak consensus on prescribing practices and a lack of evidence-based opioid management such as low utilization of multidisciplinary clinics and unfamiliarity with the WHO analgesic ladder across all specialties. The survey also indicated a lack of education regarding the prescribing of opioids across all specialties although pharmacists reported obtaining the most. Lastly, the use of risk stratification tools such as prescription drug monitoring programs and urine drug testing were underutilized amongst practitioners.

Conclusion: Strengthening practitioners’ opioid management abilities with evidence-based interventions for each aforementioned domain may aid in the fight against the opioid epidemic.

## Introduction

Opioids are commonly prescribed medications for pain management and can be an effective treatment; however, there are high risks associated with chronic opioid usage. Perceptions regarding the safety of opioids were introduced with a letter published in the New England Journal of Medicine in 1980, which reported only four instances of addiction in a review of more than 11,000 patients [1]. Several years later, a retrospective review reported addiction occurred in two of 38 patients, both of whom had a history of drug abuse [2]. This provided the grounds for a paradigm shift in how pain is managed. In a Presidential Address to the American Pain Society in 1995, the concept of “Pain as the Fifth Vital Sign” was introduced [3,4]. By 2012, healthcare providers had administered enough opioid prescriptions for every adult in the United States to have one full bottle of pills [5,6]. This surge in opioid prescriptions led to an increase in adverse effects. There was nearly a 72% increase in deaths by synthetic opioids other than methadone in 2014-15, most likely from illicitly-manufactured heroin and fentanyl in addition to a 2.6% increase from natural and semisynthetic opioids [7]. Even with a recent decline in prescribing practices, in 2017, there were noted to be 59 opioid prescriptions written for every 100 Americans [8]. Naturally, opioid administration has undergone a recent rise in scrutiny, at a local and national level.

The International Association for the Study of Pain defines pain as, “An unpleasant sensory and emotional experience associated with, or resembling that associated with, actual or potential tissue damage” [9]. Managing patients with chronic non-cancer pain then requires consideration of psychosocial aspects. This is common practice in multidisciplinary pain clinics, which utilizes a pain psychologist, social worker, pain physician, and pain therapist to help the patient understand and cope with their pain. Additionally, managing pain requires providers to monitor patients to mitigate the risks that correlate with opioid therapy such as abuse and diversion. Methods of risk stratification have been released by the CDC in 2016 for chronic non-cancer pain with the recommendation of using urine drug testing (UDT) as well as the prescription drug monitoring programs (PDMP) [5]. Education on opioid use for chronic non-cancer pain to all healthcare workers must include all the aforementioned recommendations to successfully manage patients. Each provider offers a unique amalgamation of experiences with regard to opioid prescription and stewardship that can significantly influence the number of opioids a patient is prescribed.

The perceptions and education about prescribing opioids among healthcare practitioners have not been widely studied. Currently, the level of knowledge healthcare workers have regarding these topics at a tertiary medical center is unknown. The purposes of this study are to: describe the level of knowledge providers have regarding opioid management, identify to what extent a comprehensive approach is utilized in managing patients with pain, and determine the variation in opioid prescribing habits that exist among healthcare providers. A secondary aim of this study is to investigate the knowledge and implementation of opioid management and risk stratification practices at our institution. With improved knowledge of perceptions and education among healthcare practitioners regarding their current practice of pain management, targeted education can be initiated to create a unified best practice approach for opioid utilization and prescription.

## Materials and methods

Study design

The study was submitted for the approval of the Loyola University Medical Center Institutional Review Board, Maywood, Illinois, United States, and received IRB exemption. This study was conducted by distributing a standardized survey via SurveyMonkey (Momentive Inc., San Mateo, California, United States) that was not validated due to the nature of this project being a pilot study. The initial point of distribution was by email to program directors of medical departments, residency training programs, medical education programs, and nursing education programs within a large academic institution, Loyola University Medical Center, Maywood, Illinois, United States. We consequently invited 611 physicians, 650 fellows and residents, 1073 registered nurses, 170 medical students along with nurse practitioners and pharmacists within the institution. Their participation was entirely voluntary. Data collected was in the form of a response to SurveyMonkey questions in each category. Only surveys that were fully completed were accounted for in the study, with any partially completed surveys being excluded. There was also non-identifiable demographic data collected in order to assign participants to an appropriate cohort group for further analysis.

Statistical analysis

All the questions were answerable in Likert scale scores from 1 to 5. Given the sample size and distribution, these questions were treated as continuous variables and the overall average of each group was calculated. Differences in means were evaluated in a general linear model. The global F-test assessed significant differences between all groups. If the global F-test was significant, pairwise differences were evaluated using a Šidák correction for multiple comparisons. All analyses were performed using SAS 9.4 (2013; SAS Institute Inc., Cary, North Carolina, United States) with a two-sided p-value of <.05 deemed significant.

## Results

**Figure 1:**
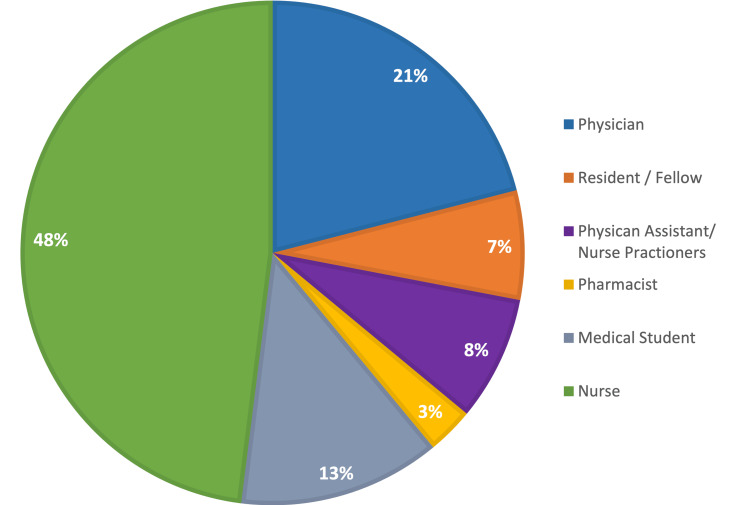
Healthcare worker participants

**Figure 2:**
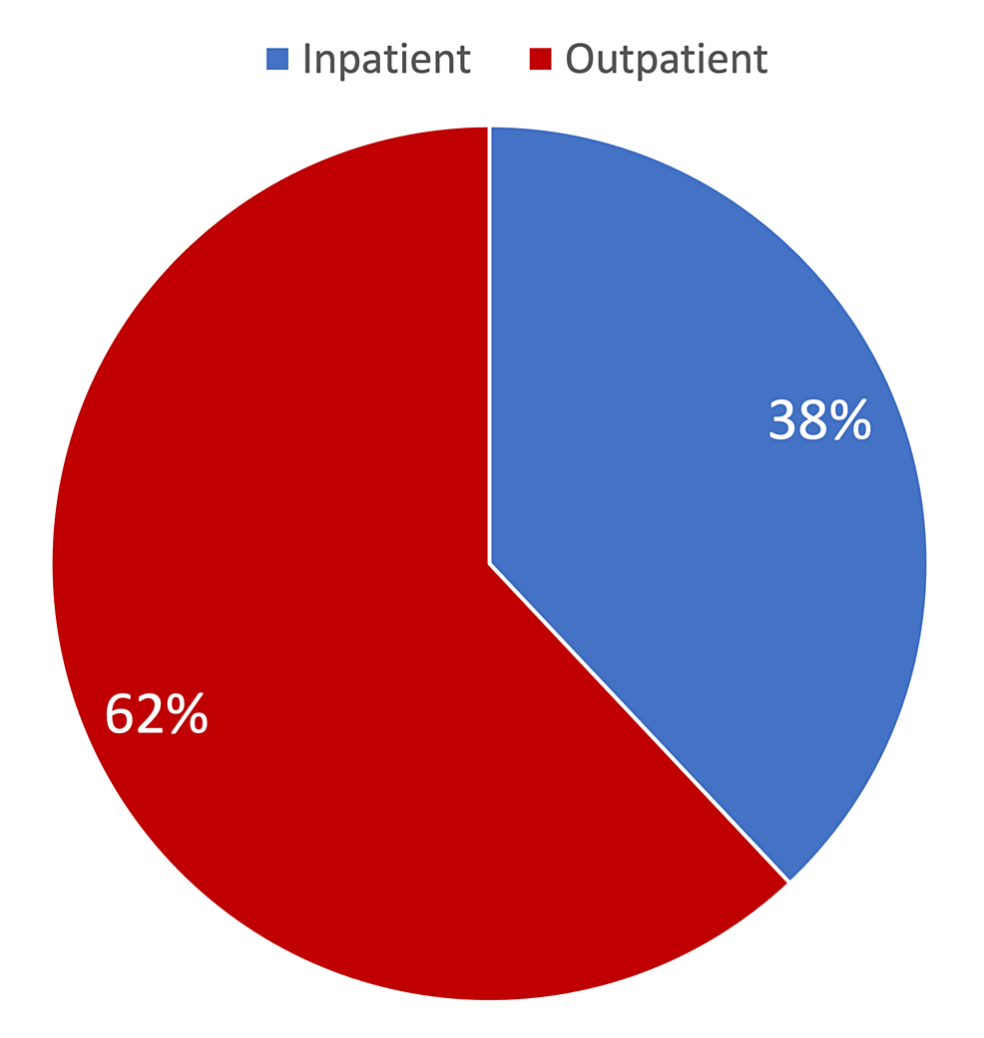
Outpatient vs. inpatient participants

A total of 638 surveys were received through SurveyMonkey. The responses consisted of 130 physicians (21%), 44 residents and fellows (6.9%), 53 physician assistants and nurse practitioners (8.31%), 18 pharmacists (2.82%), 85 medical students (13.32%), and 308 nurses (48.28%) (Figure 1). Of all participants, 61% of respondents worked primarily in an inpatient setting while 38% worked primarily in an outpatient setting (Figure 2). There were 20 specialties represented in the responses with the leading three specialties being other 15%, surgery 14%, and emergency medicine 11%. The average number of years in practice for physicians was 14 years (SD +/- 5.34) and the average number of years in practice for nursing was 12 years (SD +/- 4.29).

Prescribing habits

It was observed that physicians, physician assistants/nurse practitioners, and residents/fellows were “somewhat” to “moderately” comfortable prescribing opioids (Table 1). There were no statistically significant differences in the frequency of opioid prescribing between the providers (p = 0.3354). Nurses were significantly more likely to report higher levels of opioid prescribing than physicians (p = 0.0025)(Table 2). There were no significant differences between physicians, physician assistants/nurse practitioners, or residents/fellows with regard to frequency of referrals placed to pain management physicians; however, patients were “rarely” being referred (Table 3). Similarly, though there is no significant difference amongst providers, the utilization of multidisciplinary pain clinics was reported as only “sometimes” (p = 0.3995) (Table 4). There was also no significant difference between providers in the frequency of decreasing or discontinuing opioid therapy (p = 0.2580); however, on average, the frequency of decreasing or discontinuing opioid therapy was reported as occurring at least "sometimes" or "often" (Table 5). It was observed only “rarely” or “sometimes” that physicians, residents/fellows, or physician assistants/nurse practitioners felt pressured into prescribing opioids with no statistical difference between any group (p = 0.4969) (Table 6). Pharmacists were more likely than other providers to report that prescribing habits play a "moderate" or "significant" role in the opioid epidemic (Table 7). They were significantly more likely than physicians to attribute prescribing habits to playing a role in the opioid epidemic (p = 0.004) (Table 7).

**Table 1:** Comfortability of providers prescribing opioids *All analyses were performed using SAS 9.4 with a two-sided p-value of <0.05 deemed significant NP: nurse practitioner; PA: physician assistant

	Not Comfortable	Minimally comfortable	Somewhat comfortable	Moderately comfortable	Very comfortable				
	(1)	(2)	(3)	(4)	(5)	N	Mean	Confidence Interval	P-Value
Physicians	1	14	24	32	47	118	3.93	(3.74, 4.13)	0.3354*
NP/PA	2	5	7	21	10	45	3.71	(3.42, 4.05)	
Residents/Fellows	2	3	10	17	9	41	3.68	(3.68, 4.01)	

**Table 2:** How often do you prescribe opioids? If you are a nurse, how often do you order them? *All analyses were performed using SAS 9.4 with a two-sided p-value of <0.05 deemed significant NP: nurse practitioner; PA: physician assistant

	Never	Rarely	Sometimes	Often	Always				
	(1)	(2)	(3)	(4)	(5)	N	Mean	Confidence Interval	P-Value
Physicians	8	44	37	30	2	121	2.79	(2.63, 2.94)	0.0049*
NP/PA	5	13	6	17	5	46	3.09	(2.83, 3.34)	
Residents/Fellows	2	12	11	13	3	41	3.07	(2.80, 3.34)	
Nurses	5	21	165	43	10	244	3.13	(3.02, 3.24)	

**Table 3:** How often do you refer patients that are prescribed opioids to a pain management physician? *All analyses were performed using SAS 9.4 with a two-sided p-value of <0.05 deemed significant NP: nurse practitioner; PA: physician assistant

	Never	Rarely	Sometimes	Often	Always				
	(1)	(2)	(3)	(4)	(5)	N	Mean	Confidence Interval	P-Value
Physicians	18	27	38	30	7	120	2.84	(2.64, 3.04)	0.2587*
Residents/Fellows	9	11	13	6	2	41	2.54	(2.19, 2.88)	
NP/PA	6	8	19	11	2	46	2.89	(2.56, 3.21)	

**Table 4:** How often do you use the multidisciplinary approach to pain management? *All analyses were performed using SAS 9.4 with a two-sided p-value of <0.05 deemed significant NP: nurse practitioner; PA: physician assistant

	Never	Rarely	Sometimes	Often	Always				
	(1)	(2)	(3)	(4)	(5)	N	Mean	Confidence Interval	P-Value
Physicians	17	12	30	38	22	119	3.3	(3.08, 3.52)	0.3995*
Residents/Fellows	4	1	12	14	10	41	3.6	(3.22, 3.99)	
NP/PA	4	7	10	16	8	45	3.38	(3.01, 3.74)	

**Table 5:** How often are you decreasing or discontinuing opioid therapy? *All analyses were performed using SAS 9.4 with a two-sided p-value of <0.05 deemed significant NP: nurse practitioner; PA: physician assistant

	Never	Rarely	Sometimes	Often	Always				
	(1)	(2)	(3)	(4)	(5)	N	Mean	Confidence Interval	P-Value
Physician	11	7	32	50	19	119	3.5	(3.29, 3.70)	0.2580*
Residents/Fellows	3	7	13	16	2	41	3.17	(2.82, 3.52)	
NP/PA	7	3	10	20	6	46	3.33	(3.00, 3.65)	

**Table 6:** How often do you feel pressured into prescribing opioids? *All analyses were performed using SAS 9.4 with a two-sided p-value of <0.05 deemed significant NP: nurse practitioner; PA: physician assistant

	Never	Rarely	Sometimes	Often	Always				
	(1)	(2)	(3)	(4)	(5)	N	Mean	Confidence Interval	P-Value
Physicians	15	37	44	20	3	119	2.66	(2.47, 2.84)	0.4969*
Residents/Fellows	4	16	15	5	1	41	2.59	(2.27, 2.90)	
NP/PA	11	15	9	8	2	45	2.44	(2.14, 2.74)	

**Table 7:** How much of a role do prescribing habits have on the opioid epidemic? *All analyses were performed using SAS 9.4 with a two-sided p-value of <0.05 deemed significant NP: nurse practitioner; PA: physician assistant

	None	Minimal	Some	Moderate	Significant				
	(1)	(2)	(3)	(4)	(5)	N	Mean	Confidence Interval	P-Value
Physicians	1	10	71	38	0	120	3.22	(3.11, 3.33)	0.0034*
Residents/Fellows	0	0	23	17	0	40	3.42	(3.23, 3.62)	
NP/PA	1	2	21	22	0	46	3.39	(3.21, 3.57)	
Pharmacists	0	0	5	11	1	17	3.76	(3.47, 4.06)	

Education

There was a significant difference in the levels of formal opioid education and training reported by healthcare providers, with pharmacists reporting the highest perceived amount of education and physicians responding with the lowest (p <0.001) (Table 8). Furthermore, physicians reported significantly less formal education on opioid management than medical students (p = 0.0002) and pharmacists (p = 0.0011); residents/fellows reported significantly less education than pharmacists (p = 0.0347) and medical students reported significantly more education than nurses (p = 0.0029) (Table 8). There was a significant difference in provider familiarity with the WHO's pain management ladder (p = 0.0075), which is a framework that providers could use when developing treatment plans for cancer pain (Table 9). Pharmacists were more familiar with the WHO analgesic ladder than physicians (p = 0.0049), medical students (p = 0.002), and physician assistants/nurse practitioners (p = 0.0062) (Table 9). With regards to knowledge of utilizing a multidisciplinary approach to manage chronic pain, all providers indicated having “somewhat” familiarity, with pharmacists having the highest familiarity; however, there were no significant differences between the groups (p = 0.2351) (Table 10).

**Table 8:** How much training did you receive in medical school/residency regarding opioid management OR how much formal education have you received so far regarding opioid therapy? *All analyses were performed using SAS 9.4 with a two-sided p-value of <0.05 deemed significant NP: nurse practitioner; PA: physician assistant

	None	Minimal	Some	Moderate	Significant				
	(1)	(2)	(3)	(4)	(5)	N	Mean	Confidence Interval	P-Value
Physicians	13	38	40	24	6	121	2.77	(2.59, 2.95)	<0.001*
Residents/Fellows	3	10	17	9	2	41	2.93	(2.61, 3.23)	
Medical Students	0	10	30	28	7	75	3.43	(3.19, 3.65)	
Pharmacists	0	2	3	8	4	17	3.82	(3.34, 4.31)	
NP/PA	3	12	16	11	4	46	3.02	(2.73, 3.32)	
Nurses	24	68	88	53	15	248	2.87	(2.74, 2.99)	

**Table 9:** How familiar are you with the World Health Organization’s pain management ladder? *All analyses were performed using SAS 9.4 with a two-sided p-value of <0.05 deemed significant NP: nurse practitioner; PA: physician assistant

	Not Familiar	Minimally	Somewhat	Moderately	Very				
	(1)	(2)	(3)	(4)	(5)	N	Mean	Confidence Interval	P-Value
Physicians	29	32	31	17	12	121	2.6	(2.36, 2.82)	0.0075*
Residents/Fellows	7	9	13	7	5	41	2.85	(2.46, 3.24)	
Medical Students	19	16	17	15	8	75	2.69	(2.40, 2.98)	
NP/PA	14	8	11	8	3	44	2.5	(2.12, 2.87)	
Pharmacists	1	1	3	8	4	17	3.76	(3.15, 4.37)	

**Table 10:** How familiar are you with chronic pain management multidisciplinary programs? *All analyses were performed using SAS 9.4 with a two-sided p-value of <0.05 deemed significant NP: nurse practitioner; PA: physician assistant

	Not Familiar	Minimally	Somewhat	Moderately	Very				
	(1)	(2)	(3)	(4)	(5)	N	Mean	Confidence Interval	P-Value
Physicians	11	22	37	31	20	121	3.22	(3.02, 3.43)	0.2351*
Residents/Fellows	4	12	9	12	4	41	3.00	(2.65, 3.35)	
NP/PA	2	10	19	10	4	45	3.09	(2.75, 3.42)	
Pharmacists	0	3	4	6	4	17	3.65	(3.10, 4.20)	

Risk stratification

The final aim was to determine the frequency of urine drug screens and utilization of the PDMP to analyze the use of opioid risk stratification methods. Residents/fellows and nurses “rarely” to “sometimes” ordered urine drug screens. Physician assistants/nurse practitioners had the lowest average followed by physicians indicating they “never” or “rarely” used urine drug screens (Table 11). Physician assistants/nurse practitioners were observed to have significantly lower averages for use of urine drug screens than that of residents/fellows (p = 0.0176) and nurses (p = 0.0055) (Table 11). Physicians and physician assistants/nurse practitioners had significantly higher averages for how often they checked the PDMP compared to residents/fellows (p = 0.0038 and p = 0.001, respectively). Both physicians and physician assistants/nurse practitioners had significantly higher averages than nurses (p < 0.001) (Table 12). 

**Table 11:** How often do you or your attending physician order urine drug screens on patients taking opioids? *All analyses were performed using SAS 9.4 with a two-sided p-value of <0.05 deemed significant NP: nurse practitioner; PA: physician assistant

	Never	Rarely	Sometimes	Often	Always				
	(1)	(2)	(3)	(4)	(5)	N	Mean	Confidence Interval	P-Value
Physicians	52	37	19	9	4	121	1.98	(1.78, 2.16)	0.0008*
Resident/Fellows	9	16	8	7	1	41	2.39	(2.06, 2.71)	
NP/PA	24	15	3	4	0	46	1.72	(1.41, 2.02)	
Nurses	57	105	54	21	10	247	2.28	(2.14, 2.41)	

**Table 12:** How often do you/staff check the Illinois Prescription Monitoring Program? *All analyses were performed using SAS 9.4 with a two-sided p-value of <0.05 deemed significant NP: nurse practitioner; PA: physician assistant

	Never	Rarely	Sometimes	Often	Always				
	(1)	(2)	(3)	(4)	(5)	N	Mean	Confidence Interval	P-Value
Physicians	22	13	17	37	32	121	3.36	(3.12, 3.60)	0.001*
Residents/Fellows	10	14	7	5	5	41	2.54	(2.13, 2.94)	
NP/PA	6	4	4	13	19	46	3.76	(3.37, 4.14)	
Nurses	48	50	58	44	17	217	2.69	(2.51, 2.86)	

## Discussion

Prescribing habits

The data indicates that training and education about opioids differ among healthcare providers. Across the spectrum, physicians, physician assistants/nurse practitioners, and residents/fellows felt anywhere from “somewhat” to “moderately” comfortable prescribing opioids (Table 1). A larger cohort (39.8%) of physicians felt comfortable prescribing opioids as compared to residents (22.2%) and mid-level providers, which includes nurse practitioners and physician assistants (21.9%). Most providers note that they have received at least some degree of opioid education yet their responses regarding knowledge of the WHO analgesic ladder (Table 9) and chronic pain multidisciplinary clinics (Table 10) are much varied. This contrast in responses demonstrates a disconnect between opioid education and prescribing habits. Surveyed healthcare providers believe that prescribing opioids does, at least in part, contribute to an opioid epidemic. Conversely, pharmacists feel that those that prescribe opioids play a stronger role in the opioid epidemic (Table 7).

The majority of healthcare providers demonstrated “somewhat” of a familiarity with the WHO analgesic ladder (Table 9). Interestingly, however, a large proportion of all prescribers (physicians, residents, and midlevel providers) had “minimal” to “no” familiarity with the WHO analgesic ladder (50.4%, 39%, and 50%, respectively). Of all pharmacists surveyed, on the other hand, only 11.7% had “minimal” to “no” familiarity with the WHO analgesic ladder, with the vast majority being “moderate” to “very” familiar with the WHO ladder. Though primarily used for cancer pain, the WHO analgesic ladder has proven efficacy in pain management and has been repeatedly demonstrated to be a primary means of opioid stewardship and reduction [10,11]. It can be inferred from these findings that pharmacists’ have a stronger knowledge of the pharmacotherapy and management of opioids.

The results also demonstrate a disparity among providers regarding the prescription of multidisciplinary treatment modalities for pain management and referrals to a pain management specialist (Tables 4, 10). A larger cohort of physicians was unaware or had “never” utilized these services for their patients. A multidisciplinary approach to pain management was observed to be utilized “sometimes” for all groups with no significant differences between prescribers. Further, a referral to a pain management specialist was observed to be “rarely” or “sometimes” for all groups (Tables 4, 10). Multidisciplinary pain clinics have been repeatedly cited to promote overall opioid reduction while providing long-term pain relief [12]. The results illustrate a contrast in that providers feel comfortable prescribing opioid medications, yet they are unaware of the most up-to-date and effective means of managing patients’ opioid prescriptions. This data offers a future direction in which subsequent intervention could help target these practices.

Opioid education

The results support previously reported literature, that there is a lack of education regarding how to properly prescribe opioids [13-15]. However, there does appear to be a shift as medical students were noted to have statistically significantly more education regarding opioids than physicians. In 2018, the Association of American Medical Colleges found that 87% of 102 medical schools examined had curricula dedicated to pain and substance use in the context of pain [16]. However, 97% of respondents reported challenges in educating students on opioids. The most frequent challenge noted was “the need for greater faculty and resident expertise to teach and model safe prescribing practices'' [16]. Despite these challenges, there appears to be a general trend of more knowledge of opioids in those with fewer years of training, indicating that education is beginning at earlier stages of medical training. However, given there was no difference in knowledge of the WHO analgesic ladder nor utilization of multidisciplinary clinics (Tables 4, 10), it is clear more education on pain management for medical students, residents, and physicians is necessary. Presently, there is no standardization of what a comprehensive education on opioids should include in medical institutions [16]. There should be a standardization of pain management curricula including the WHO analgesic ladder, the importance of utilizing pain specialists and multidisciplinary clinics when clinically indicated, and utilizing risk stratification methods.

The data suggests that pharmacists are more knowledgeable in these subjects suggesting that they have received more adequate training regarding opioids. According to several studies, pharmacists typically counsel patients on the safe use of their opioid medication and possible side effects; however, they feel they have inadequate training, poor communication with providers, and lack of access to adequate health information to counsel appropriately [17,18]. Based on the results, it could be hypothesized that increased pharmacist involvement could improve prescribing habits of their physician counterparts. Some pharmacists and physicians have developed drug therapy management teams or collaborative practice models in which both practitioners share accountability [19]. A further study into pharmacist training and education is warranted. If there are discrepancies in education between pharmacists and healthcare providers, then it would become worthwhile to integrate a pharmacist-driven curriculum into physician education of opioids, thereby improving opioid knowledge.

Risk stratification

All healthcare professionals could benefit from additional training in how to appropriately manage pain with opioids including content on how to stratify risk. Residents had the highest average for utilizing drug screens and physicians had the lowest. It can be inferred, therefore, that residents compared to physicians have stronger drug screening knowledge, particularly since these guidelines are relatively new. In 2016, the CDC released its Opioid Prescribing Guidelines and recommended the use of UDT upon opioid therapy initiation and annually thereafter to assess prescription and illicit drug use [5]. Numerous studies have shown that consistent UDT implementation has moderate efficacy in improving opioid adherence [20] and prescription safety [21,22], though consistent methodology between studies is lacking. Regardless, UDT provides a means of early opioid aberrancy detection that could influence treatment modifications and result in better outcomes. The study indicated that these guidelines were not observed in the majority of providers surveyed (Table 11). Any unexpected UDT results should not result in the dismissal of care and instead should be used as a means to educate patients and improve safety. Mitigating risk during opioid therapy is essential to lessen the effects of the opioid crisis. Physicians should consider the positive effect UDT can have on patient safety and their prescribing habits.

In addition to UDT, the CDC gives a grade “A” recommendation for the use of PDMPs [5]. According to the results, physicians and physician assistants/nurse practitioners utilize the PMDP more than residents but still, its implementation averaged from “rare” to “sometimes”. PDMP use has been shown to improve prescribing habits in healthcare settings such as the ED by decreasing inappropriate opioid prescribing [23]. Further, as individual states release their PDMP data, reductions in average opioid dispensing and dosage have decreased as well as the number of individuals who have visited five or more prescribers seeking opioids in the past 90 days [24]. As electronic medical records are currently integrating PDMP into their interfaces, and as states are requiring providers to interact with PDMP, the use of the program and the data it produces will become more evident. The study indicated that PDMP utilization was highest among physicians and physician assistants/nurse practitioners relative to other healthcare providers. In contrast, the data showed that residents and nurses on average checked the PDMP significantly less. Part of this might be explained by the fact that actual prescribers might be more inclined to check the information PDMPs provide to influence their prescribing habits. The other part might be explained by recent policy changes at this academic institution, which now requires prescribers to check the PDMP prior to prescribing. Consequently, the data may be directly related to the implementation of this mandatory requirement. Yet as the opioid epidemic continues, it only seems appropriate that the fight against it is multifactorial. Though nurses and residents are not prescribing, their use of the PDMP could work as a safety net to ensure appropriate drug administration [25]. The information presented within the PDMP consequently appears to be an integral piece for any healthcare worker.

Future direction

Understanding opioid perceptions among healthcare providers can enable practitioners in an academic hospital to improve communication, implement multidisciplinary and pain specialist referrals, and increase overall opioid education. The study was able to establish specific interventions as indicated in Table 13. Devising a tailored curriculum for healthcare professionals can accomplish the primary goal of more effective opioid management. Furthermore, awareness of opioid stewardship lends itself to improved risk stratification for all patients through UDT and PDMP screening. By capturing the various perceptions of opioid management within a healthcare system, it stands to reason that as practitioners become more aware of their differences, the more unified their approach can be in appropriate opioid management. 

**Table 13:** Future recommendations CME: continuing medical education; UDT: urine drug testing; PDMP: prescription drug monitoring programs

	Intervention
Prescribing habits/ practice management	Recommend an increased role of pharmacists in caring for individuals using opioids, increased utilization of multidisciplinary pain clinics and pain specialist referrals, utilization of the WHO pain ladder
Education	Create a standardized pain management curriculum to include the WHO analgesic ladder, emphasize utilizing pain specialists when clinically indicated, and utilizing risk stratification methods, include pharmacists as guest lecturers at grand rounds and other resident/healthcare providers' educational activities to broaden scope of information given; hospital-driven initiative provides online CME courses for all healthcare providers annually to improve opioid knowledge
Risk stratification	Administer validated risk assessment tools to ensure a more objective approach to assessing a patient's opioid risk. Using this assessment, we can prioritize specific tools such as UDT to minimize the patient’s risk. All associated healthcare providers should be involved in checking the PDMP in patients receiving opioid prescriptions to minimize error

Limitations of the study

A limitation of the study is that it is a single-institution study; there is sampling bias, and as such may not be applicable to all other institutions. Additionally, some survey questions were not answered by all individuals, thereby showing a slight discrepancy between data sets. The study being strictly a survey, there is a recall bias given participants may not accurately remember the amount of education focused on opioids they received during their training. Lastly, there may be variability between specialty familiarity in prescribing opioids, specifically among those practicing in surgical versus non-surgical specialties.

## Conclusions

Understanding opioid perceptions among healthcare workers can enable practitioners in an academic hospital to improve communication, implement multidisciplinary and pain specialist referrals, and increase overall opioid education. The data from this survey-based study indicate providers are “somewhat” comfortable prescribing opioids but that their training and education about opioids differ. It was noted that there is a general trend that medical students are receiving more education in opioids than physicians have in the past and that pharmacists overall have the most education regarding opioids. The results also demonstrate a disparity among providers regarding the prescription of multidisciplinary treatment modalities for pain management and referrals to a pain management specialist. Lastly, the data suggests underutilization of the PDMP and UDTs.

Based on the results of this study, the following recommendations can be made to improve opioid prescribing practices: increasing the role of pharmacists in caring for individuals using opioids, increasing utilization of multidisciplinary clinics and pain specialist referrals, increasing utilization of the WHO pain ladder, urine drug screens, and the PDMP, and through the creation of standardized pain management curriculum for medical students, provide online continuing medical education (CME) courses for all healthcare providers annually to improve opioid knowledge.
